# Holopelagic *Sargassum* aggregations provide warmer microhabitats for associated fauna

**DOI:** 10.1038/s41598-023-41982-w

**Published:** 2023-09-13

**Authors:** Alexandra G. Gulick, Nerine Constant, Alan B. Bolten, Karen A. Bjorndal

**Affiliations:** 1 Archie Carr Center for Sea Turtle Research and Department of Biology, University of Florida, Gainesville, FL USA; 2 Present Address: Glacier Bay National Park and Preserve, National Park Service, Bartlett Cove, AK USA

**Keywords:** Ecology, Ocean sciences, Marine biology

## Abstract

Drifting aggregations of *Sargassum* algae provide critical habitat for endemic, endangered, and commercially important species. They may also provide favorable microclimates for associated fauna. To quantify thermal characteristics of holopelagic *Sargassum* aggregations, we evaluated thermal profiles of 50 aggregations in situ in the Sargasso Sea. Sea surface temperature (SST) in the center of aggregations was significantly higher than in nearby open water, and SST differential was independent of aggregation volume, area, and thickness. SST differential between aggregation edge and open water was smaller than those between aggregation center and aggregation edge and between aggregation center and open water. Water temperature was significantly higher inside and below aggregations compared to open water but did not vary inside aggregations with depth. Holopelagic *Sargassum* aggregations provide warmer microhabitats for associated fauna, which may benefit marine ectotherms, though temperature differentials were narrow (up to 0.7 °C) over the range of aggregation sizes we encountered (area 0.01–15 m^2^). We propose a hypothetical curve describing variation in SST differential with *Sargassum* aggregation size as a prediction for future studies to evaluate across temporal and geographic ranges. Our study provides a foundation for investigating the importance of thermal microhabitats in holopelagic *Sargassum* ecosystems.

## Introduction

*Sargassum* is a diverse genus of brown macroalgae found in tropical to temperate marine environments worldwide^1^. All but two of > 350 *Sargassum* species are benthic and spend at least part of their life cycle attached to the substrate, forming underwater canopies from which biomass may detach and disperse^1^. *Sargassum natans* and *S. fluitans* are holopelagic species that remain in a free-floating state for their entire life cycle, drifting at the sea surface and reproducing asexually through fragmentation^1–3^. Holopelagic *Sargassum* (subsequently referred to as *Sargassum*) is restricted to the Atlantic Ocean^1^ and supports a unique floating ecosystem in the pelagic zone^3–5^.

*Sargassum* distribution at broad scales is driven by surface currents and winds, as well as spatiotemporal variation in growth and mortality^6,7^. Historically, *Sargassum* was most abundant within the North Atlantic Gyre in an area of open ocean in the western Atlantic known as the Sargasso Sea^2,3^. Based on surface net tows in 1933–1935, *Sargassum* density in the Sargasso Sea was over four times greater than in the Gulf of Mexico and over forty times greater than in the Caribbean Sea^2^. Satellite imagery from 2003 to 2008 revealed a pattern of seasonal export of *Sargassum* from the Gulf of Mexico into the western Atlantic, and *Sargassum* biomass in the Gulf of Mexico and Sargasso Sea were estimated at one million metric tons each^6^. Beginning in 2011, *Sargassum* abundance increased dramatically in the tropical Atlantic, associated with *Sargassum* proliferation in the North Equatorial Recirculation Region and accompanied by large masses of *Sargassum* accumulating on the coasts of Caribbean and West African countries^6,8^. *Sargassum* blooms in this region have recurred nearly annually since 2011^9,10^. In 2018, *Sargassum* biomass in the “Great Atlantic *Sargassum* Belt” extending from West Africa through the Caribbean into the Gulf of Mexico exceeded twenty million metric tons^9^.

Considerable attention has focused on the proliferation of *Sargassum* in the tropical Atlantic, resulting in a growing body of work aimed at understanding driving factors (e.g. refs.^9–11^) and improving forecasting of beaching events (reviewed in ref.^10^), as well as identifying approaches to ameliorating negative socioeconomic and environmental impacts of nearshore and beached *Sargassum* (e.g. refs.^12,13^). Faunal communities associated with *Sargassum* in the tropical Atlantic and Caribbean Sea have received less attention^14,15^. Because in-water removal of *Sargassum* is an emerging management approach, recent studies emphasize the need for an improved understanding of the role *Sargassum* plays in supporting biodiversity^16^. Continued investigation of the habitat function of *Sargassum* remains important given the ongoing change in *Sargassum* biomass^10^ and well-established ecological value of *Sargassum* in its historical range^4,5,17^.

Termed “hedgerows of the epipelagic environment” by Archie Carr due to their importance for marine life^18^, aggregations of *Sargassum* are hubs of biodiversity in the open ocean and provide critical habitat for endemic, endangered, and commercially important species^4,5,17^. At least ten endemic species are associated with *Sargassum* among > 145 invertebrate, > 127 fish, and four sea turtle species that use this habitat in the Atlantic^4,5,17,19^. Where structure is otherwise limited, *Sargassum* aggregations offer refuge from predation, shelter from water movement, and productive foraging and nursery habitat^4,5,19–21^. *Sargassum* aggregations may also provide warmer environments for associated fauna by absorbing solar energy and reducing water movement and heat dispersal^22,23^, thereby increasing water temperature relative to open water.

Macroalgal structure can be a source of thermal heterogeneity at the microhabitat scale, and, in some cases, creates favorable thermal microclimates for associated species^24–26^. Microclimate, or temperature in the immediate surroundings of an organism^27^, directly impacts performance, fitness, and thermoregulatory behavior of ectotherms and fish^28–31^. We expect that holopelagic *Sargassum* may serve a similar function for associated fauna. Airborne thermal infrared imagery^22^ and ex situ temperature measurements of seawater with and without *Sargassum*^23^ support the prediction that *Sargassum* aggregations are warmer than open water. However, thermal profiles of *Sargassum* aggregations have not been evaluated in situ, and knowledge of thermal characteristics of *Sargassum* aggregations is otherwise limited.

To better understand the habitat function of holopelagic *Sargassum* and evaluate whether *Sargassum* aggregations provide warmer microhabitats for associated fauna, we addressed the following objectives in situ in the Sargasso Sea: (1) compare sea surface temperature (SST) in the center of *Sargassum* aggregations and in nearby open water; (2) evaluate the relationship between aggregation dimensions and SST differential between aggregation center and open water; and (3) quantify water temperature gradients inside and below aggregations and compare them to nearby open water. We provide a foundation for evaluating thermal profiles of *Sargassum* aggregations across temporal and geographic continua and for investigating the importance of thermal microhabitats for *Sargassum*-associated fauna.

## Methods

### Study area and sampling design

**Figure 1:**
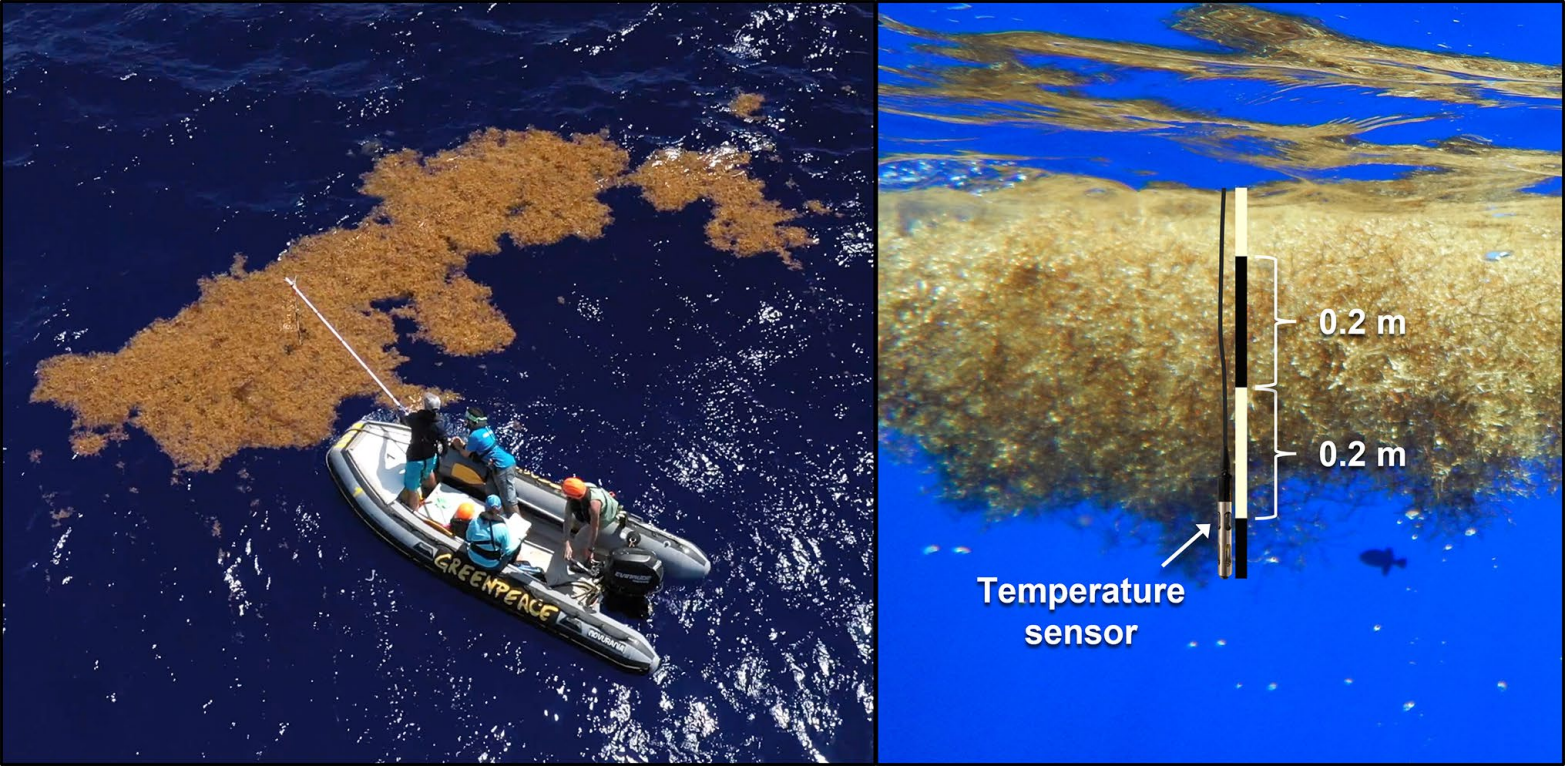
Researchers measure water temperatures in a *Sargassum* aggregation using a YSI Pro20i temperature probe rigged to an extendable pole (left), using black bands on the PVC pipe as a depth reference (right). Photo credits: Tavish Campbell/Greenpeace (left); N.C. (right).

We evaluated thermal profiles of 50 *Sargassum* aggregations by assessing temperature differentials between aggregations and nearby open water during a research expedition to the Sargasso Sea from 30 July to 12 August 2019 aboard the Greenpeace vessel MV *Esperanza*. After departing from Bermuda, vessel headings were set using ocean surface temperature and ocean color imagery obtained from Roffer’s Ocean Fishing Forecasting Service (https://www.roffs.com) to target convergence zones and increase the probability of encountering *Sargassum* aggregations. All sampling occurred southeast of Bermuda (30.3–31.5°N, 61.7–63.9°W) within the boundaries of the Sargasso Sea (22–38°N, 43–76°W) defined by the clockwise flow of major ocean currents^5^. Daily, from 0800 to 1800 h, observers with binoculars on the outer bridge wings of the vessel (circa 11.3 m above sea level) monitored the port and starboard sides of the vessel for *Sargassum*. When *Sargassum* was sighted, a rigid inflatable boat (RIB) was deployed from which to conduct sampling (Fig. 1).

Sampling was conducted during daylight hours when the solar disk was not obstructed by clouds and in sea states of Beaufort Force ≤ 3. Sampling was restricted to these conditions to minimize variation in incoming solar radiation and water movement and control for temporal variation in environmental conditions that affect seawater temperature^32^ and *Sargassum* aggregation state^33^. Drifting *Sargassum* occurs as dispersed fragments and individual thalli or “clumps”^17,21,33,34^ typically 0.1–0.5 m across^3^; lines or “windrows” of *Sargassum* aggregated parallel with the wind direction and stabilized by Langmuir circulation^2,3^ varying in width from < 0.5 m to several m^22,33,34^; and “mats” or “rafts” of aggregated *Sargassum* several m in extent up to tens or hundreds of m across^3,17,22,33,34^, which may be associated with windrows^33,34^ or occur as discrete aggregations more likely to form in calm conditions^2,3^. *Sargassum* distribution in the Sargasso Sea is irregular and patchy^3^, large aggregations are uncommon, and clumps and windrows are most frequently observed in groups of 1–5^33^. Because patterns of *Sargassum* aggregation and distribution observed during the study period were consistent with previous reports, we sampled *Sargassum* aggregations as they were encountered unless aggregations of multiple size strata were visible from the RIB. If so, to ensure the range of aggregation sizes sampled represented the range of aggregation sizes present, we sampled aggregations ≥ 1 m wide (n = 28) as they were encountered and sampled every fourth aggregation < 1 m wide (n = 22) encountered. *Sargassum* aggregations were considered independent units if they were at least 5 m apart.

Aggregation length and maximum width (perpendicular to the length axis) were visually estimated to the nearest 0.25 m from an eye height above sea level of 2.0 m. Using a YSI Pro20i temperature probe rigged to an extendable pole (Fig. 1), we measured SST at 0.5-m intervals along a transect from aggregation edge at the aggregation’s widest point toward aggregation center. At each horizontal position, we also inserted the probe into the aggregation to measure temperature at 0.2-m increments to a depth of 1 m. After recording temperature measurements, we snorkeled into the aggregation to measure aggregation thickness at each horizontal position using a PVC pole marked at 0.2-m increments. For aggregations < 0.5 m wide, only SST at aggregation center was measured, and thickness was visually estimated to the nearest 0.1 m. For aggregations ≥ 0.5 and < 1 m wide, only temperatures at edge and center were measured, and length and width were recorded to the nearest 0.05 m. For each aggregation, we measured temperatures at 0.2-m depth increments, as described above, in nearby open water at a single point circa 5 m away. For aggregations in which we only measured SST, only SST was measured at the paired open water point. Using an extendable pole (up to 4.9 m) allowed for data collection across the entire range of aggregation sizes encountered while minimizing water disturbance by the RIB. See Supplementary Information for a video of our methods.

Because the greater proportion of aggregations were elongate (length > width), we calculated area for each aggregation as maximum width × length rather than as circular area. We calculated aggregation volume as area × thickness at aggregation center. We classified temperature measurements as inside aggregation or below aggregation if depth of the measurement was ≤ or > aggregation thickness, respectively.

### Statistical analyses

Analyses were performed in R v. 4.0.0^35^ using the dplyr package^36^. SST in the center of *Sargassum* aggregations was compared to nearby open water using a non-parametric paired-samples Wilcoxon test. Linear regression was used to evaluate relationships between aggregation dimensions (volume, area, thickness) and SST differential (aggregation center–open water), with log-transformed (dimension) and (differential + 1) to meet parametric assumptions. Non-parametric Kruskal–Wallis and post-hoc Dunn’s tests with Bonferroni correction were used to compare SST differentials among open water, aggregation edge, and aggregation center; temperatures inside aggregations, below aggregations, and open water; and temperatures among 0.2-m depth intervals. Differences in sample size among groups are due to taking fewer temperature measurements for smaller aggregations, as described above. The assumption of normality was checked for all comparisons using Shapiro–Wilk tests. Homogeneity of variance was checked using Levene’s tests for comparisons with a categorical predictor variable and by visual inspection of residual plots for comparisons with a continuous predictor variable. When parametric assumptions were not met, a non-parametric alternative was used, as specified above. All statistical tests were two-tailed, and significance was evaluated at an alpha level of 0.05. All means are reported ± standard error (s.e.m.).

## Results

**Figure 2:**
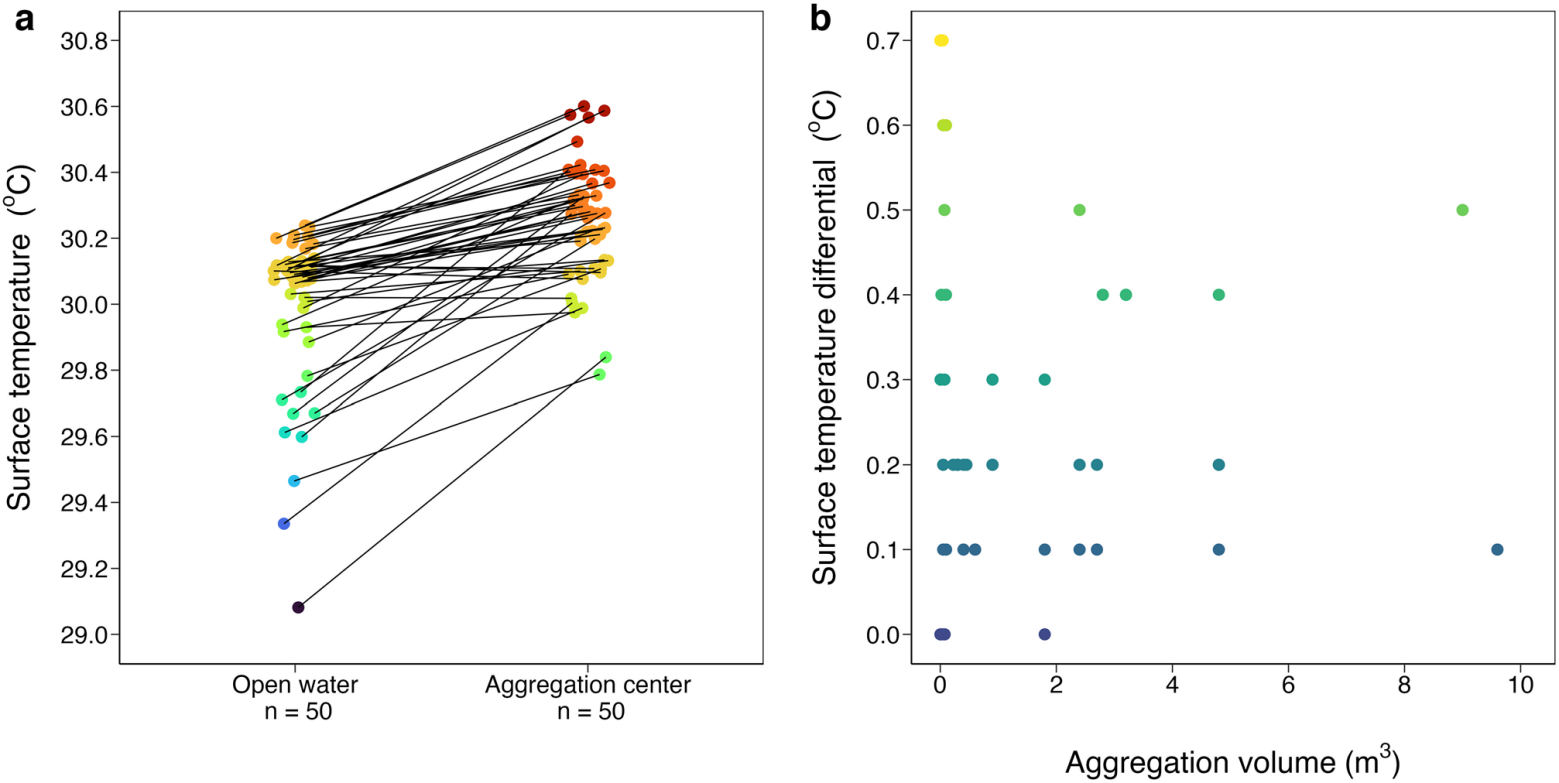
(**a**) SST at *Sargassum* aggregation center (30.3 °C ± 0.03) was significantly higher than in nearby open water (30.0 °C ± 0.03; n = 50, V = 0, *p* < 0.01). Connecting lines indicate paired water temperature measurements. (**b**) SST differential between aggregation center and open water was independent of aggregation volume (n = 48, F_1,46_ = 2.09, *p* = 0.16). Respective color scales for surface temperature and temperature differential match the corresponding y-axes and are consistent among Figs. 2, 3, 4, 5.

Water temperature was measured between 1100 and 1800 h in *Sargassum* aggregations (n = 50) ranging in width (0.1–3.0 m, median 0.75 m), area (0.01–15 m^2^, median 1.3 m^2^), thickness (0.1–1.4 m, median 0.4 m), and volume (0.001–9.6 m^3^, median 0.4 m^3^) (Supplementary Fig. S1). SST in the center of *Sargassum* aggregations (30.3 °C ± 0.03) was significantly higher than in nearby open water (30.0 °C ± 0.03) (n = 50, V = 0, *p* < 0.01; Fig. 2a). Range in SST differential (0.0–0.7 °C) corresponds to a temperature range in open water of 29.1–30.2 °C and in aggregation center of 29.8–30.6 °C. SST differential between aggregation center and open water was independent of aggregation volume (n = 48, F_1,46_ = 2.09, *p* = 0.16; Fig. 2b), area (n = 50, F_1,48_ = 1.76, *p* = 0.19; Supplementary Fig. S2a), and thickness (n = 48, F_1,46_ = 2.80, *p* = 0.10; Supplementary Fig. S2b). Though SST differential did not correlate with aggregation dimensions, the highest temperatures (≥ 30.6 °C) were recorded in larger aggregations (area ≥ 6 m^2^; Supplementary Fig. S3, Table S1).

**Figure 3:**
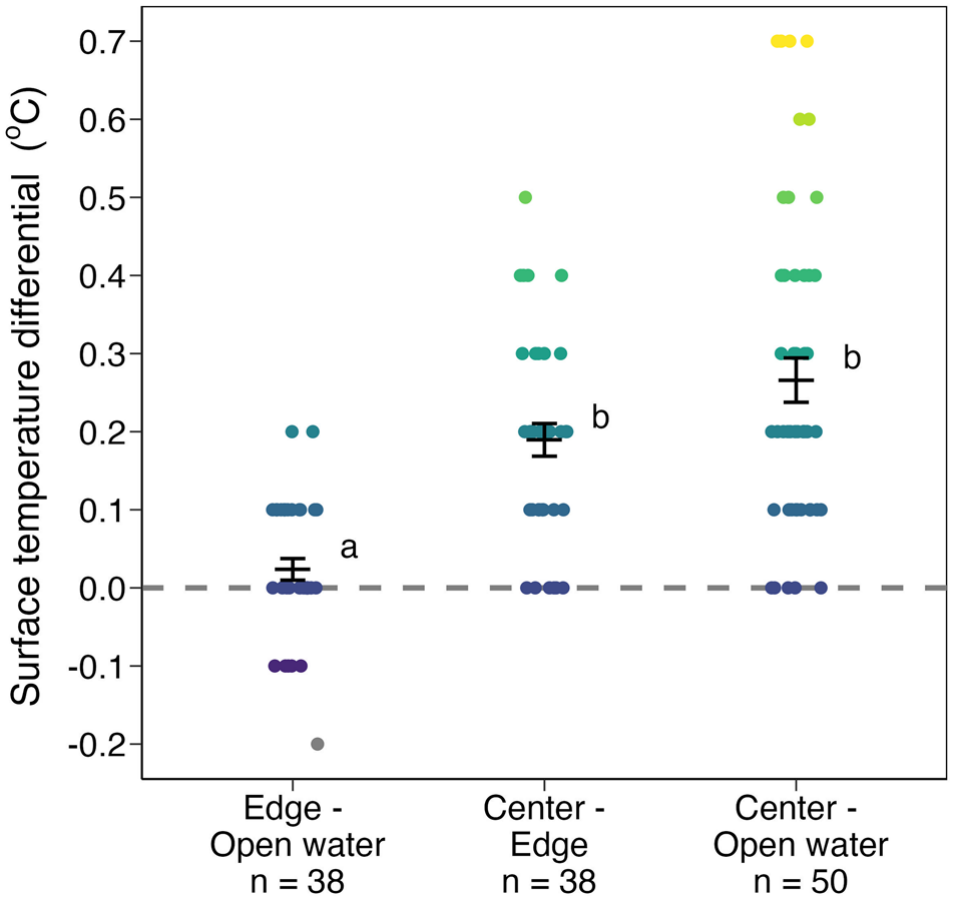
SST differential between *Sargassum* aggregation edge and open water (n = 38, 0.02 °C ± 0.01) was smaller than differentials between aggregation center and aggregation edge (n = 38, 0.19 °C ± 0.02) and between aggregation center and open water (n = 50, 0.27 °C ± 0.03; d.f. = 2, H = 47.06, *p* < 0.01). Black bars are means ± s.e.m. Shared lowercase letters indicate significant post-hoc pairwise comparisons (*p* < 0.05; Table 1).

**Figure 4:**
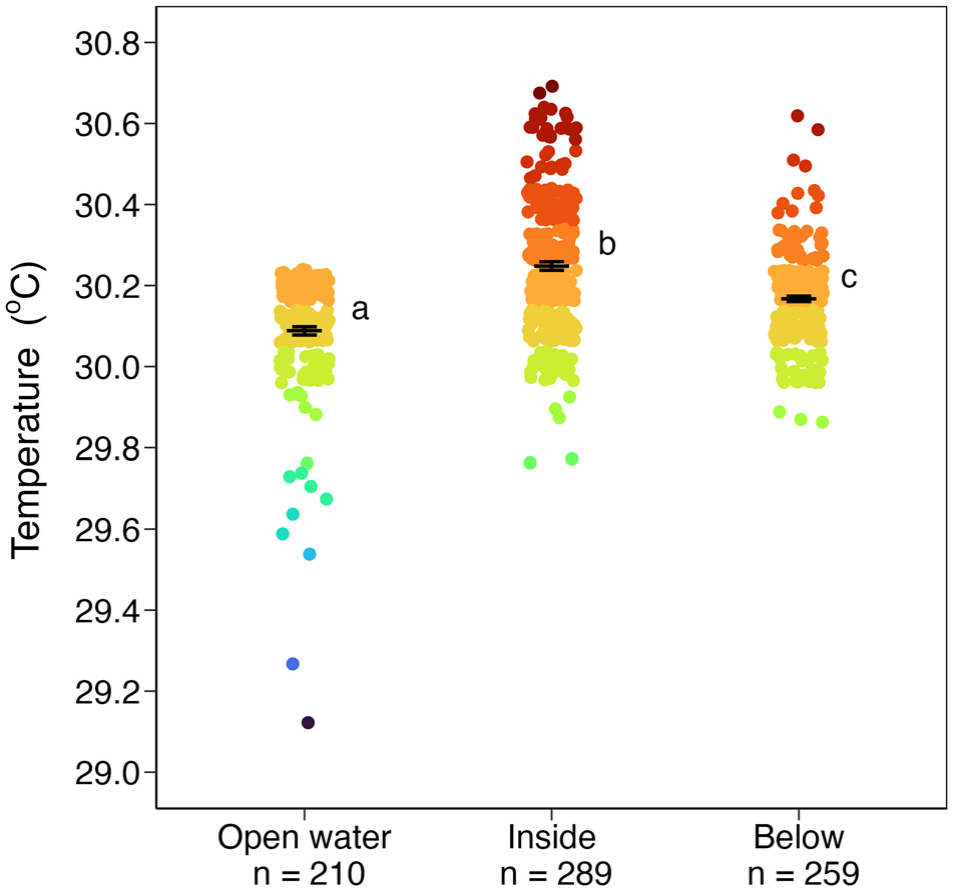
Water temperature was significantly higher inside (30.25 °C ± 0.01) and below (30.17 °C ± 0.01) *Sargassum* aggregations compared to nearby open water (30.09 °C ± 0.01; d.f. = 2, H = 102.53, *p* < 0.01). Black bars are means ± s.e.m. Shared lowercase letters indicate significant post-hoc pairwise comparisons (*p* < 0.05; Table 2).

**Figure 5:**
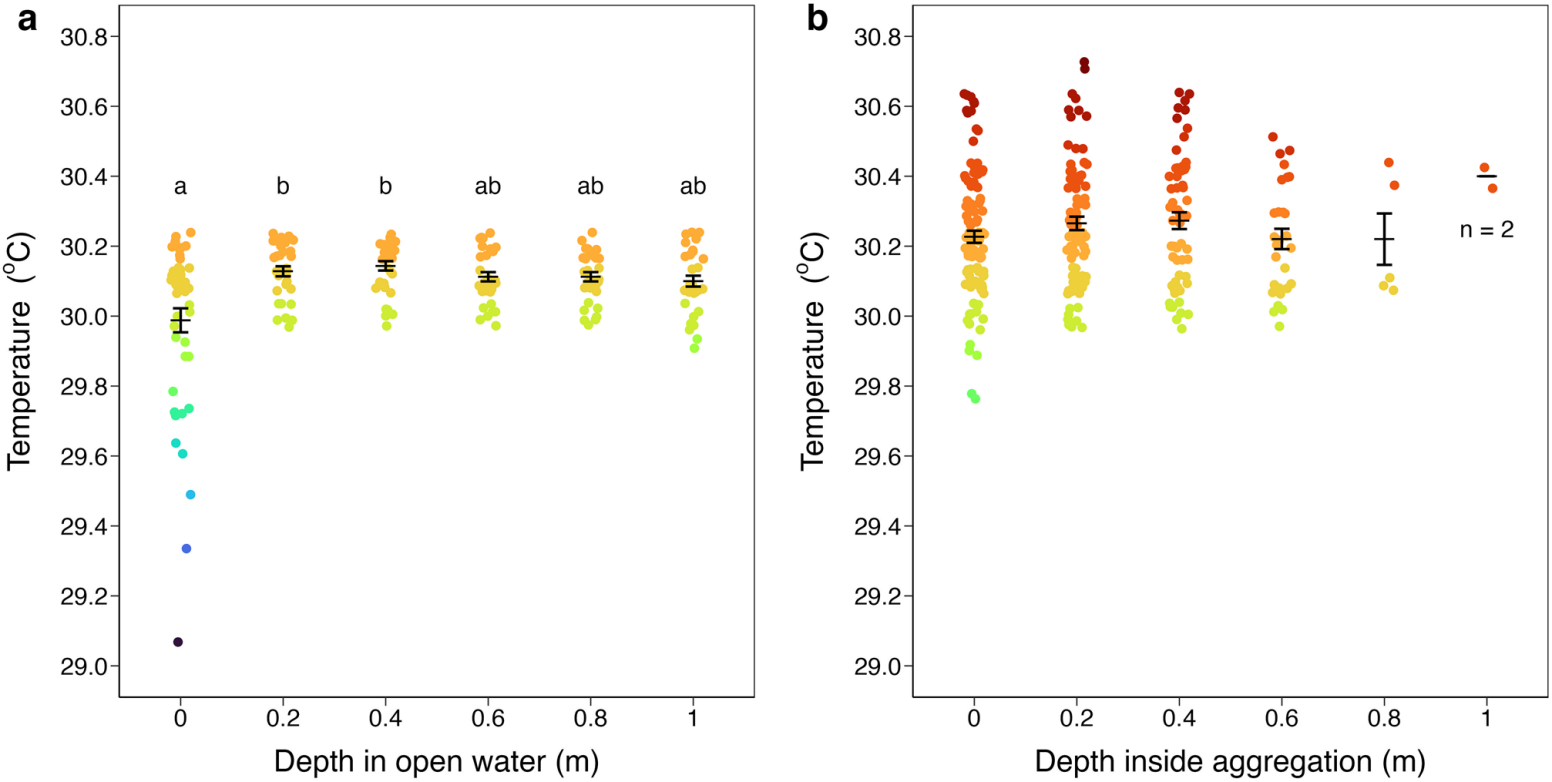
(**a**) Temperature in open water varied significantly with depth (d.f. = 5, H = 19.44, *p* < 0.01). Open water temperature at the surface (n = 50, 29.99 °C ± 0.03) was lower than at depths of 0.2 m (n = 32, 30.13 °C ± 0.01; Z = 3.17, adjusted *p* = 0.023) and 0.4 m (n = 32, 30.14 °C ± 0.01; Z = 4.03, adjusted *p* < 0.01). (**b**) Water temperature inside aggregations did not vary significantly with depth (d.f. = 4, H = 3.54, *p* = 0.47; depth for which n < 5 excluded from comparison). Black bars are means ± s.e.m. Shared lowercase letters indicate significant post-hoc pairwise comparisons.

SST differential between aggregation edge and open water (n = 38, 0.02 °C ± 0.01) was smaller than the differential between aggregation center and aggregation edge (n = 38, 0.19 °C ± 0.02) and smaller than the differential between aggregation center and open water (n = 50, 0.27 °C ± 0.03) (d.f. = 2, H = 47.06, *p* < 0.01; Fig. 3, Table 1). Water temperature was significantly higher inside (n = 289, 30.25 °C ± 0.01) and below (n = 259, 30.17 °C ± 0.01) *Sargassum* aggregations compared to open water (n = 210, 30.09 °C ± 0.01) (d.f. = 2, H = 102.53, *p* < 0.01; Fig. 4, Table 2; Supplementary Fig. S4). Water temperature varied significantly with depth in open water (d.f. = 5, H = 19.44, *p* < 0.01; Fig. 5a) but not with depth inside *Sargassum* aggregations (d.f. = 4, H = 3.54, *p* = 0.47; Fig. 5b).

**Table 1:** Post-hoc pairwise comparisons of SST differentials (°C) among open water, *Sargassum* aggregation edge, and aggregation center using Dunn's test with Bonferroni correction.

Group	n	Mean	s.e.m
Edge–Open water	38	0.02	0.01
Center–Edge	38	0.19	0.02
Center–Open water	50	0.27	0.03

Comparison		Z	Adjusted *p*
Edge–Open water	Center–Edge	5.19	< 0.01
Edge–Open water	Center–Open water	6.56	< 0.01
Center–Edge	Center–Open water	1.02	0.92

**Table 2:** Post-hoc pairwise comparisons of temperature (°C) in open water, inside *Sargassum* aggregations, and below aggregations using Dunn's test with Bonferroni correction.

Group	n	Mean	s.e.m
Open water	210	30.09	0.01
Inside	289	30.25	0.01
Below	259	30.17	0.01

Comparison		Z	Adjusted *p*
Open water	Inside	10.1	< 0.01
Open water	Below	5.31	< 0.01
Inside	Below	−4.95	< 0.01

## Discussion

### Factors driving temperature differentials

We show that *Sargassum* aggregations ≤ 3.0 m wide are warmer than nearby open water. Previous work has stimulated interest in thermal characteristics of *Sargassum* aggregations^22^ and the potential benefit of this thermal niche for associated organisms^23^. Our study is the first to evaluate thermal profiles of this habitat in situ and at a scale relevant to conditions experienced by *Sargassum*-associated fauna.

Light attenuation due to high macrophyte^37–39^ or phytoplankton biomass^40^ results in surface water warming. *Sargassum* aggregations likely affect water temperature through the same mechanism^22,23^, altering the absorption profile of solar radiation^41,42^. Temperature differentials are likely driven by increased heating of water inside *Sargassum* aggregations mediated by environmental factors (incoming solar radiation, wind speed, current velocity) and aggregation characteristics like size and density^37,38,40^. *Sargassum* aggregations range in size from clumps < 0.5 m across to dense mats hundreds of m across^2,3,33,34^. Although SST differential did not correlate with aggregation dimensions over the range of aggregation sizes we encountered, results from remote sensing of seawater temperature^22^ support the prediction that temperature differential will vary with aggregation size and suggest there may be a size threshold above which temperature differential increases. Marmorino et al.^22^ reported that *Sargassum* aggregations > 10 m across (area > 80 m^2^) appeared 0.1–0.5 °C warmer than adjacent seawater in airborne imagery, and a differential was not detected for smaller aggregations. Our study provides direct field observations supporting solar warming of smaller *Sargassum* aggregations (area ≤ 15 m^2^).

**Figure 6:**
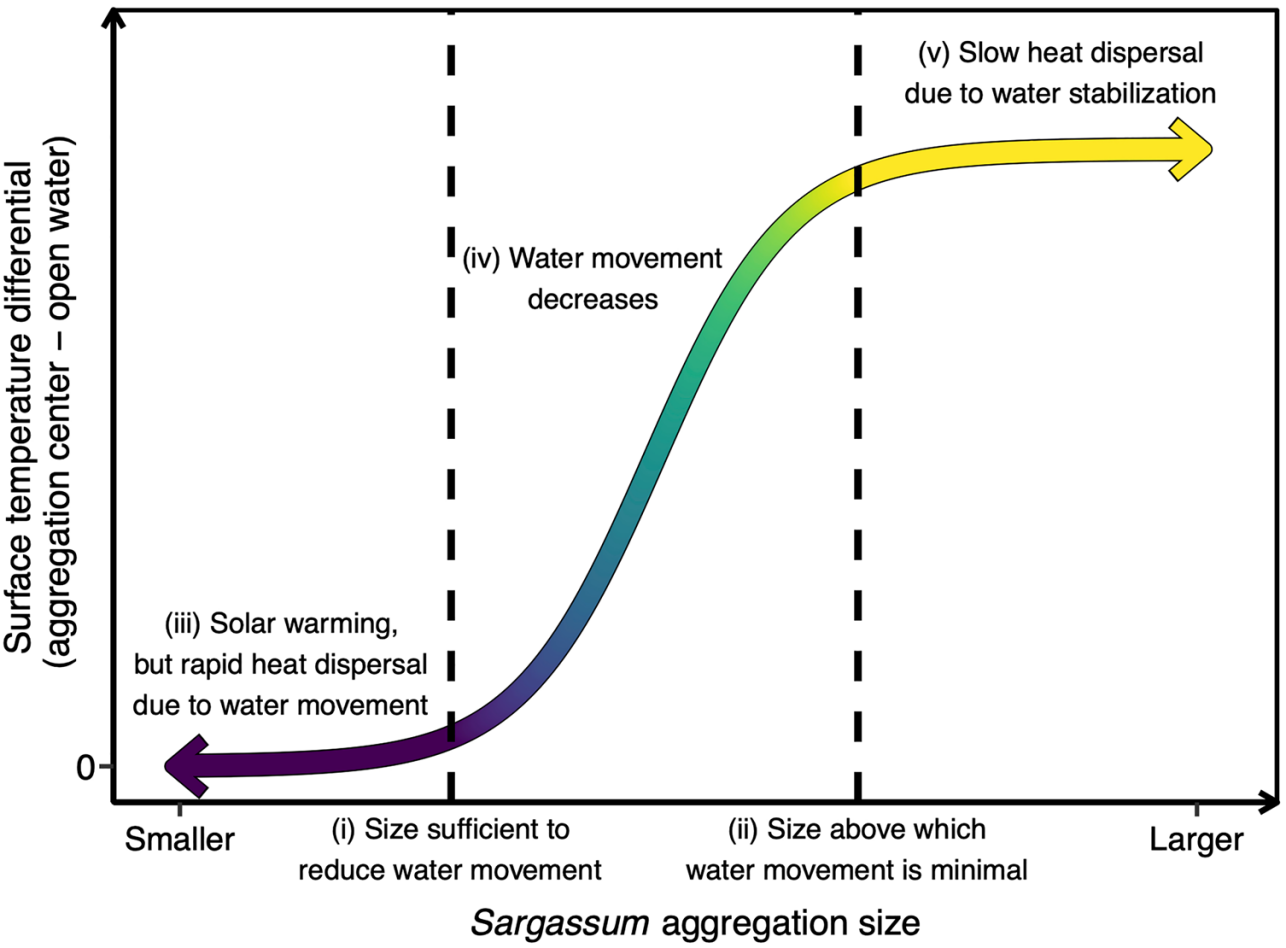
Predicted effect of *Sargassum* aggregation size on the surface temperature differential between *Sargassum* aggregation center and open water.

The range in aggregation size sampled in the current study is consistent with the aggregation characteristics most frequently encountered in the Sargasso Sea^3,33^ and in the tropical Atlantic and Caribbean Sea^33,34^. Future studies should quantify thermal characteristics of larger *Sargassum* aggregations in situ, given continued expansion of *Sargassum* biomass^10^ and the recurrence of *Sargassum* aggregations > 50 m across in the Caribbean and off West Africa^33,34^. Though the geographic scope of sampling in the current study was limited to an area southeast of Bermuda in the Sargasso Sea, solar warming of the ocean surface occurs in all ocean basins^32^, thus we expect the mechanisms driving warming of *Sargassum* aggregations are consistent within and among regions. We predict SST differential increases with aggregation size above a threshold sufficient to reduce water movement inside the aggregation (Fig. 6(i)), with the greatest increase in temperature relative to open water at the center of aggregations large enough to minimize water movement and slow heat dispersal (Fig. 6(ii)).

Water movement driven by currents and wind could substantially affect temperature differentials in two ways. First, water movement through aggregations may transport warmed water out and cooler water in. Natural water movement, apparent in our methods video (see Supplementary Information), likely increased water transport and reduced temperature differentials in the current study (Fig. 6(iii–iv)). Second, water movement and wind acting directly on emergent *Sargassum* alter shapes and densities of *Sargassum* aggregations^3,22^, thereby affecting thermal stability, which we predict would be greater in large, dense aggregations (Fig. 6(v))^38,41,43^. *Sargassum* aggregations have been described as “wave-subduers”^44^. The stabilizing effect of *Sargassum* may be responsible for reduced water temperature variance at the surface of aggregations (Fig. 5b) compared to SST in open water (Fig. 5a) potentially via reduced air-sea heat flux^32^.

Diel variation in SST^32^ may also affect temperature differentials. In the Sargasso Sea, diurnal SST ranges 0.1–2.6 °C during the diurnal thermal cycle, with minimum and maximum temperatures occurring at 0400–0500 and 1500–1600 UT, respectively^45^. Diurnal temperature fluctuation in dense benthic *Sargassum* canopies lags hours behind water outside the canopy, with daytime temperatures maintained inside canopies at night^46,47^. This was recorded in a *Sargassum* forest 50 m wide with a canopy height of 3 m^47^, which is comparable to the dimensions of the largest holopelagic *Sargassum* aggregations reported in the eastern Caribbean (50–100s of m wide and up to 7 m thick)^34^. Thus, it is unlikely that temperatures in typical *Sargassum* aggregations (up to several m wide^33,34^ and ≤ 0.5 m thick^34^), including those sampled in the current study, lag behind open water. Diurnal variation in temperature differentials should be evaluated across depth in larger *Sargassum* aggregations. In dense macrophyte canopies, daytime temperatures are cooler and more stable below the surface, while at night, surface water cooling combined with reduced water movement in dense structure can cause temperature to be higher with depth^43^. Greater thermal stability in large *Sargassum* aggregations should result in slower warming after sunrise than in open water and daytime temperature differentials that decrease with depth, as well as longer retention of daytime heat after sunset and nighttime temperature differentials that increase with depth.

### Biological significance of Sargassum microclimates

Temperature affects the rate at which biochemical reactions occur, and metabolic rates scale with temperature^30,48,49^. Across diverse taxa and habitats, most rates of biological activity increase exponentially with temperature over the temperature range in which an organism is normally active^30^. Small marine ectotherms, which dominate the holopelagic *Sargassum* community^4,20^, have body temperatures that track ambient water temperature^29^. Thermal microclimates directly impact ectotherm performance and influence fitness^29,30^. Fish and other mobile ectotherms are able to regulate body temperature by moving among thermal microhabitats^28,31^. Behavioral thermoregulation can reduce metabolic costs, improve performance, and increase fitness^28, 50^. Similar to other aquatic macroalgae^24–26^, holopelagic *Sargassum* should provide favorable thermal microclimates for associated fauna^23^. Due to a lack of knowledge related to species thermal tolerance ranges for those associated with holopelagic *Sargassum*, it is difficult to assess how species’ thermal tolerance coincides with the temperature differentials we documented in *Sargassum* aggregations, or how climate change may affect the viability of thermal microclimates.

Rising atmospheric CO_2_ concentrations are resulting in ocean warming and increased acidity^51–53^. The complexity of factors mediating thermal thresholds and fitness outcomes^54–56^, as well as limited knowledge of species’ thermal tolerance ranges, genetic adaptation potentials, and acclimation abilities^57,58^, make predicting impacts of warming on individual species difficult. Under future climate conditions, temperatures in holopelagic *Sargassum* aggregations would exceed upper thermal limits of associated species before temperatures in open water. If selecting *Sargassum* habitat becomes maladaptive despite other fitness advantages^58^, *Sargassum* aggregations may become ecological traps. Tropical species are probably more vulnerable to warming than temperate species because most have narrower thermal tolerance breadths and limited acclimation ability^56,58–60^. Thus, risk must be evaluated across latitudes in which holopelagic *Sargassum* is found. Predicting the effects of climate change on thermal microhabitats of holopelagic *Sargassum* aggregations is outside of the scope of our study. However, our theoretical model of the relationship between temperature differential and aggregation size (Fig. 6) provides a conceptual framework for exploring potential impacts of ocean warming on thermal microclimates, in addition to evaluating thermal profiles across broader geographic and temporal continua.

Because temperature differentials have important biological implications for *Sargassum* ecosystems, future studies should evaluate temporal and geographic variation in thermal microhabitats in situ, particularly of larger aggregations. Our study provides a foundation for investigating the importance of thermal microclimates for holopelagic *Sargassum*-associated fauna, which has important implications for anticipating impacts of climate change on the suitability of this thermal habitat for endemic, endangered, and commercially important species that depend on *Sargassum* ecosystems.

### Supplementary Information

Supplementary Information 1.

Supplementary Information 2.

Supplementary Information 3.

Supplementary Information 4.

## Data Availability

The data and code supporting the current study are available in the Dryad Digital Repository (10.5061/dryad.1vhhmgr07)^61^.
