# Supplementary material for: Holopelagic Sargassum aggregations provide warmer microhabitats for associated fauna
Source: Sci Rep. 2023 Sep 13;13:15129. doi: 10.1038/s41598-023-41982-w (PMC10499784; doi:10.1038/s41598-023-41982-w)
Supplement: Supplementary file 1 — Supplementary Information 1. [file 41598_2023_41982_MOESM1_ESM.pdf]

Scientific Reports

Supplementary Information

Holopelagic *Sargassum* aggregations provide warmer microhabitats for associated fauna

Alexandra G. Gulick[1,2,*,†], Nerine Constant[1,*,†], Alan B. Bolten[1], Karen A. Bjorndal[1]

[1] Archie Carr Center for Sea Turtle Research and Department of Biology, University of Florida, Gainesville, Florida, U.S.A.

[2] Current location: Glacier Bay National Park and Preserve, National Park Service, Bartlett Cove, AK, U.S.A.

[*] Corresponding authors: alexandra.gulick@ufl.edu & nconstant@ufl.edu

[†] Equal first authorship

Supplementary Methods

Video of A.G.G. and N.C. evaluating thermal profiles of *Sargassum* aggregations *in situ*, including aerial footage of the following:

00:00 Measuring water temperatures in *Sargassum* aggregations using a YSI Pro20i temperature probe rigged to an extendable pool pole (up to 4.9 m), which allowed for data collection while minimizing disturbance to aggregation thermal environment by the rigid inflatable boat. Black marks at 0.2-m increments on the PCV pipe were used as a depth reference when inserting the temperature probe into the aggregation to measure temperature at 0.2-m depth increments to a depth of 1 m. Video clips show different viewing angles of two aggregations.

00:34 After recording temperature measurements, N.C. snorkels into an aggregation to measure aggregation thickness from edge to center using a PVC pipe marked at 0.2-m increments. A.G.G. remains at aggregation edge to collect video footage.

01:18 Environmental conditions in the Sargasso Sea representative of the study period. Sampling was conducted during daylight hours when the solar disk was not obstructed by clouds and in sea states of Beaufort Force ≤3. The 72.3-m Greenpeace research vessel, MV *Esperanza*, is visible in the background.

Video credit: Tavish Campbell / Greenpeace

Supplementary Results

Tables

Table S1. Summary of water temperature measurements in open water and across *Sargassum* aggregation horizontal (distance into aggregation) and vertical (depth) dimensions (visualized in Fig. S3). Mean temperature and standard error (s.e.m.) are reported if n > 1.

Distance into aggregation (m)	Depth (m)	n	Temperature (°C)	s.e.m.
Open water	0 (Surface)	50	29.99	0.034
	-0.2	32	30.13	0.014
	-0.4	32	30.14	0.013
	-0.6	32	30.11	0.013
	-0.8	32	30.11	0.013
	-1	32	30.10	0.016
0 (Edge)	0	34	30.12	0.017
	-0.2	8	30.06	0.026
0.05	0	1	30.30	-
0.075	0	1	30.10	-
0.125	0	2	29.95	0.150
0.15	0	2	30.05	0.050
0.2	0	1	30.40	-
0.225	0	2	29.90	0.100
0.25	0	4	30.23	0.048
0.3	0	2	30.20	0.000
0.35	0	2	30.30	0.000
0.375	0	1	30.10	-
0.5	0	23	30.31	0.038
	-0.2	21	30.32	0.044

	-0.4	11	30.21	0.049
	-0.6	2	30.30	0.100
	-0.8	1	30.40	-
	-1	1	30.40	-
0.75	0	6	30.32	0.031
	-0.2	5	30.36	0.040
	-0.4	1	30.40	-
1	0	11	30.35	0.041
	-0.2	9	30.34	0.044
	-0.4	7	30.33	0.061
	-0.6	2	30.25	0.150
	-0.8	1	30.40	-
	-1	1	30.40	-
1.5	0	6	30.20	0.077
	-0.2	6	30.22	0.087
	-0.4	4	30.10	0.071
2	0	9	30.36	0.073
	-0.2	9	30.37	0.075
	-0.4	5	30.22	0.058
	-0.6	1	30.10	-
2.5	0	1	30.60	-
	-0.2	1	30.60	-
	-0.4	1	30.60	-

Figures

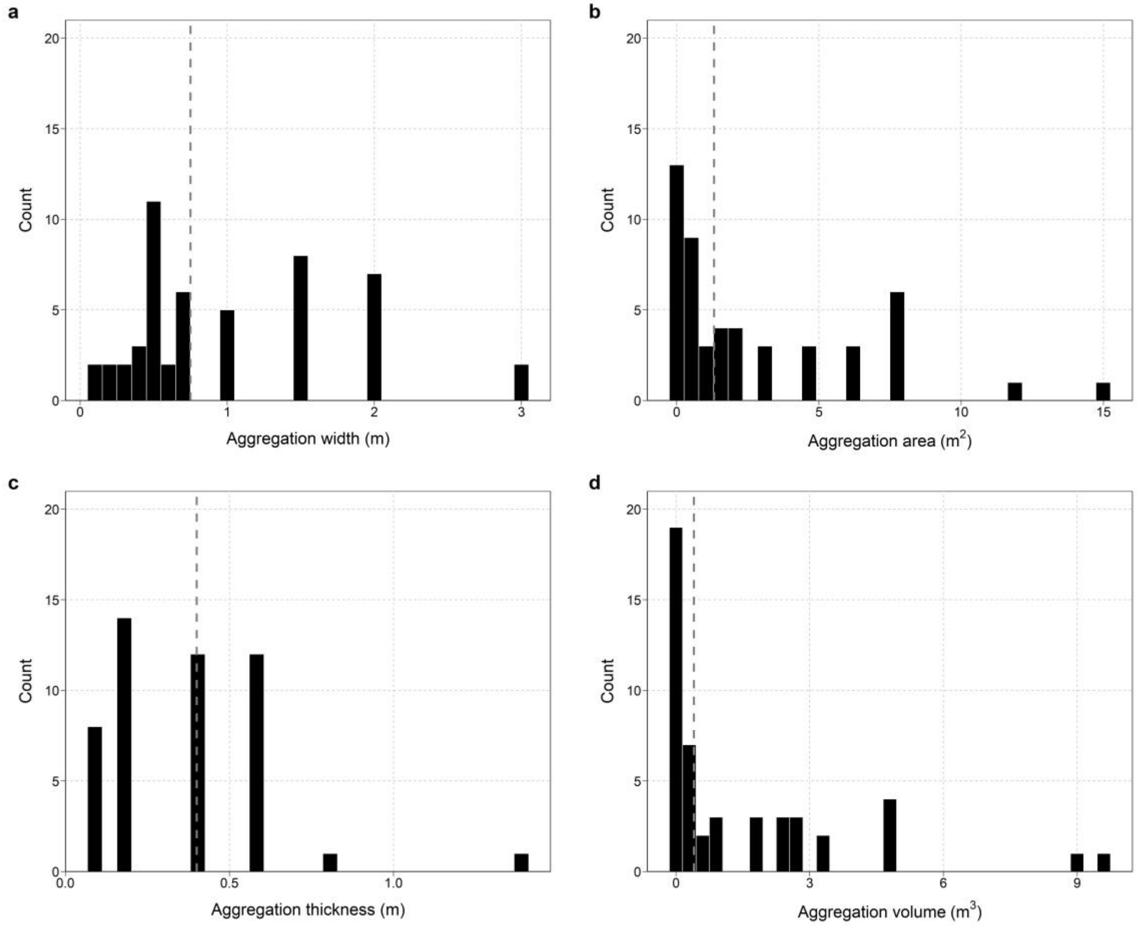

Figure S1. Temperature was measured in *Sargassum* aggregations (n = 50) ranging in (**a**) width (0.1–3.0 m, median 0.75 m), (**b**) area (0.01–15 m^2, median 1.3 m^2), (**c**) thickness (0.1–1.4 m, median 0.4 m), and (**d**) volume (0.001–9.6 m^3, median 0.4 m^3). Thick dashed line indicates the median. Aggregation thickness was not measured for n = 2 aggregations, so n = 48 for thickness and volume.

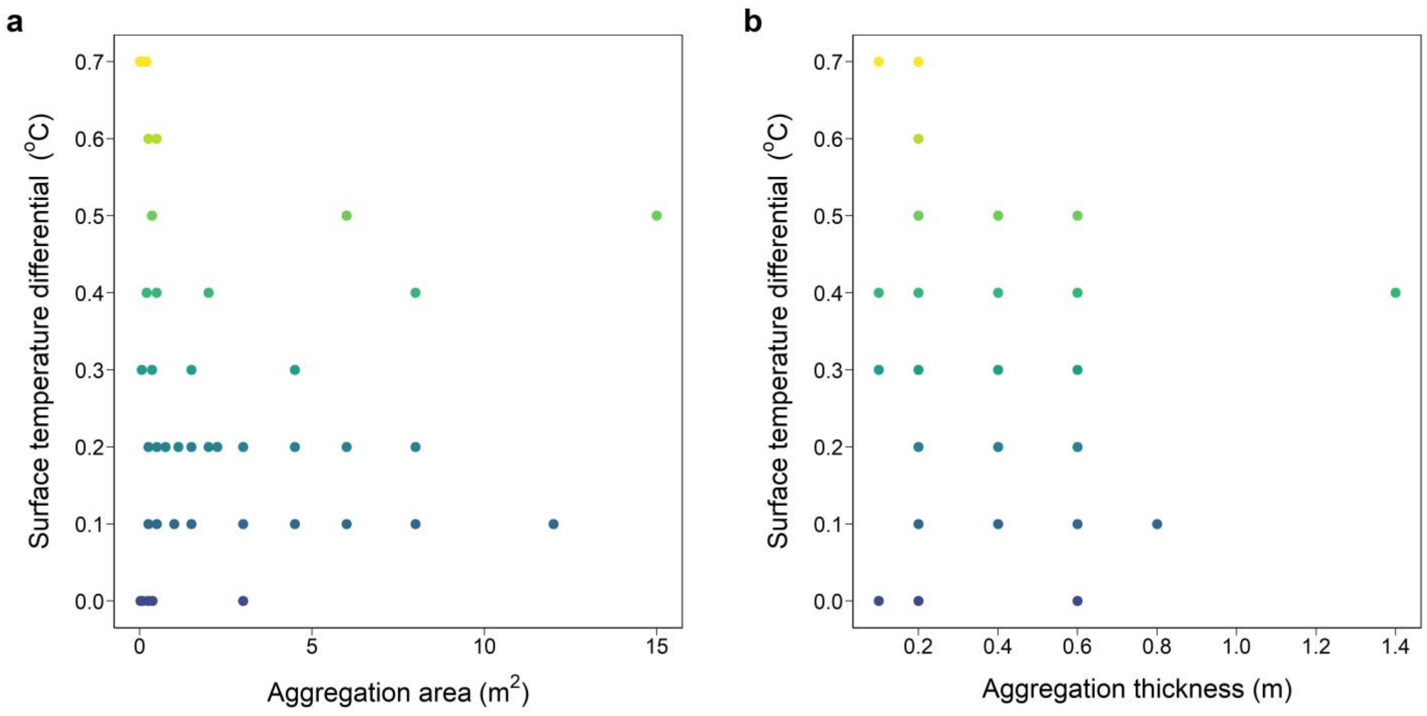

Figure S2. SST differential between *Sargassum* aggregation center and nearby open water was independent of (**a**) area (n = 50, $F_{1,48}$ = 1.76, p = 0.19) and (**b**) thickness (n = 48, $F_{1,46}$ = 2.80, p = 0.10) across the range of aggregation sizes encountered. Linear regressions of x = log(x) and y = log(y + 1). The range in SST differential (0.0–0.7 °C) corresponds to a temperature range in open water of 29.1–30.2 °C and in mat center of 29.8–30.6 °C. Color scale for temperature differential matches the y-axis and is consistent among figures.

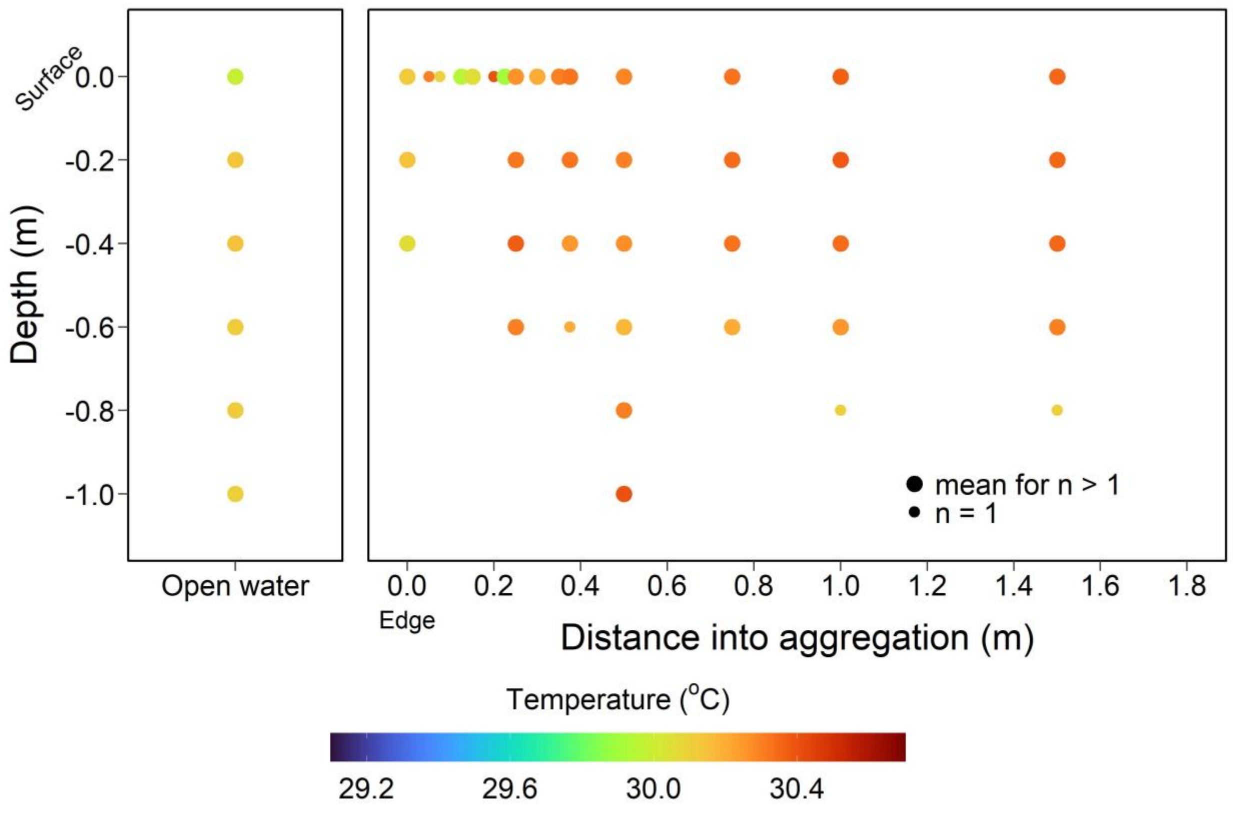

Figure S3. Heatmap of temperature measurements in nearby open water and across *Sargassum* aggregation horizontal (distance into aggregation) and vertical (depth) dimensions (summarized in Table S1). Measurements were taken at 0.2-m increments of depth and either at 0.5-m intervals of distance from aggregation edge to center (aggregations ≥1 m wide), at edge and center (aggregations <1 and ≥0.5 m wide), or only at aggregation center (aggregations <0.5 m wide). Distance into aggregation values at aggregation center were calculated as $\frac{aggregation\ width}{2}$. The highest temperatures (30.6–30.7°C) were recorded in larger aggregations (area ≥6 m^2; 80th percentile), though SST differential did not correlate with aggregation dimensions over the range of *Sargassum* aggregation sizes we encountered (Fig. S2; main text Fig. 2b).

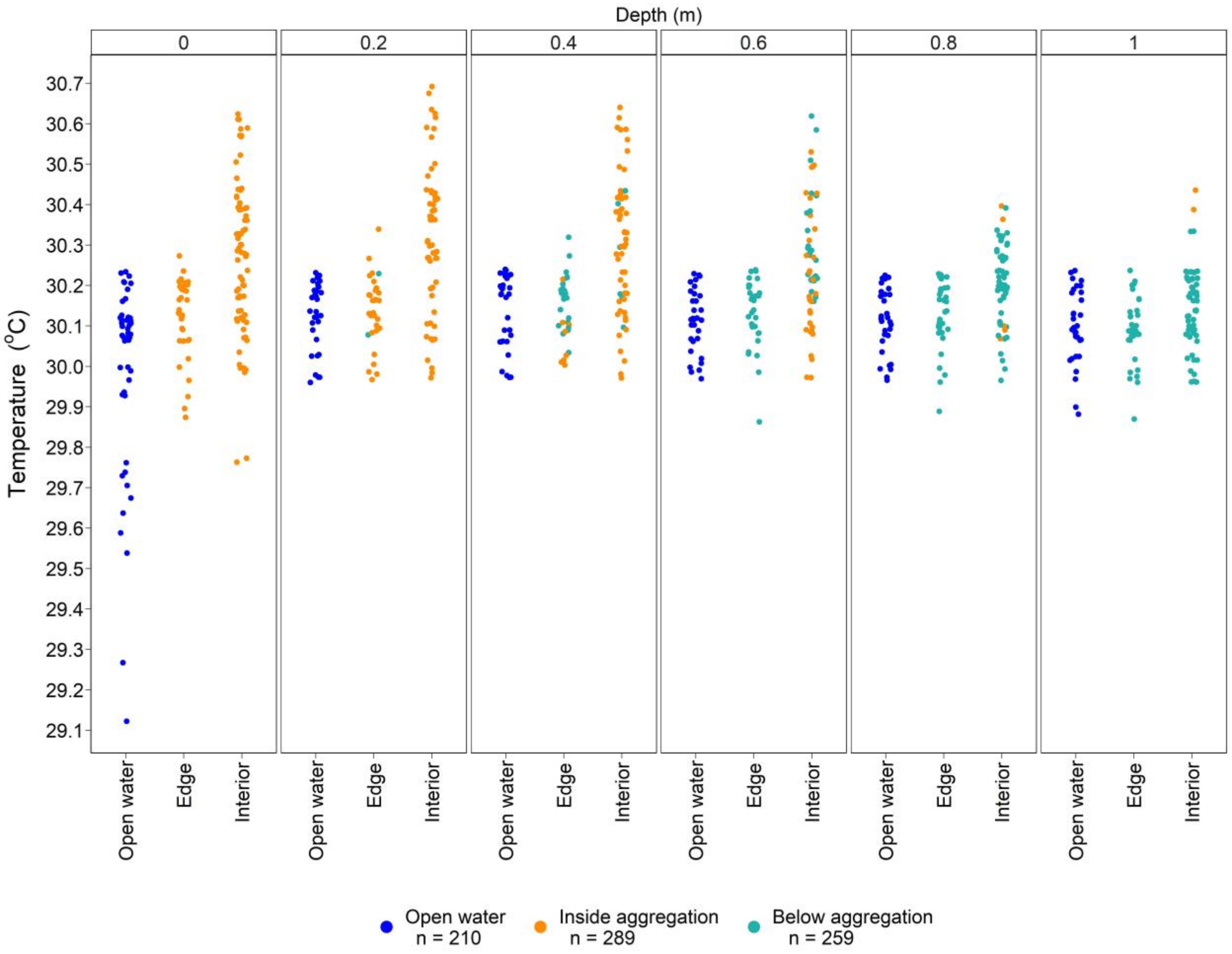

Figure S4. Water temperature across the range of locations (open water, inside *Sargassum* aggregation, below *Sargassum* aggregation) and depth (from sea surface down to 1 m) sampled in our study. We classified temperature measurements as inside aggregation or below aggregation if depth of the measurement was ≤ or > aggregation thickness, respectively. Edge and interior refer to the horizontal position at which temperature was measured (edge at 0 m distance into aggregation; interior at >0 m, including at aggregation center).
